# 7-Aminoalkoxy-Quinazolines from Epigenetic Focused Libraries Are Potent and Selective Inhibitors of DNA Methyltransferase 1

**DOI:** 10.3390/molecules27092892

**Published:** 2022-04-30

**Authors:** José L. Medina-Franco, Edgar López-López, Liliam P. Martínez-Fernández

**Affiliations:** 1 DIFACQUIM Research Group, Department of Pharmacy, School of Chemistry, National Autonomous University of Mexico, Mexico City 04510, Mexico; elopez.lopez@cinvestav.mx (E.L.-L.); liliammtzfdz@gmail.com (L.P.M.-F.); 2 Department of Pharmacology, Center for Research and Advanced Studies of the National Polytechnic Institute (CINVESTAV), Mexico City 07360, Mexico

**Keywords:** docking, drug discovery, enzyme inhibition, epigenetics, epi-informatics, focused library, molecular dynamics, multi-target epigenetic agent, polypharmacology, quinazoline

## Abstract

Inhibitors of epigenetic writers such as DNA methyltransferases (DNMTs) are attractive compounds for epigenetic drug and probe discovery. To advance epigenetic probes and drug discovery, chemical companies are developing focused libraries for epigenetic targets. Based on a knowledge-based approach, herein we report the identification of two quinazoline-based derivatives identified in focused libraries with sub-micromolar inhibition of DNMT1 (30 and 81 nM), more potent than *S*-adenosylhomocysteine. Also, both compounds had a low micromolar affinity of DNMT3A and did not inhibit DNMT3B. The enzymatic inhibitory activity of DNMT1 and DNMT3A was rationalized with molecular modeling. The quinazolines reported in this work are known to have low cell toxicity and be potent inhibitors of the epigenetic target G9a. Therefore, the quinazoline-based compounds presented are attractive not only as novel potent inhibitors of DNMTs but also as dual and selective epigenetic agents targeting two families of epigenetic writers.

## 1. Introduction

The identification of drug candidates targeting epigenetic targets is of large interest for addressing several therapeutic needs [1,2]. Several epi-drugs currently approved for clinical have been reviewed elsewhere [3]. Similarly, it is attractive identifying tool compounds to understand better epigenetic processes. Among the epigenetic drug and probe candidates, small molecules targeting epigenetic writers such as DNA methyltransferases (DNMTs) and protein lysine methyltransferases (PKMTs) are promising for the treatment of various types of cancer such as colorectal, breast, lung, ovarian, pancreatic cancer and acute myeloid leukemia [4,5,6,7,8], neurological disorders [9,10], autoimmune diseases [11,12], and metabolic diseases [13,14,15].

DNA methylation is mediated by the enzyme family DNMTs that is responsible for catalyzing the covalent addition of a methyl group from *S*-adenosyl-*L*-methionine (SAM) to the 5-carbon of cytosine, mainly within CpG dinucleotides, yielding *S*-adenosyl-*L*-homocysteine (SAH). DNMT3A and DNMT3B are maintenance meanwhile DNMT1 is a de novo methyltransferase. DNMT3A is overexpressed in vulvar squamous cell carcinoma and pituitary adenoma [16] however, it is mainly associated with hematological malignancies [17]. DNMT3B is overexpressed in lung, ovarian, and breast cancer, hepatocellular carcinoma, and mild traumatic brain injury [17]. DNMT1 is associated with colorectal, pancreatic, gastric, lung, and thyroid cancer and pituitary adenoma, lupus [18], and hereditary sensory neuropathy [19].

**Figure 1:**
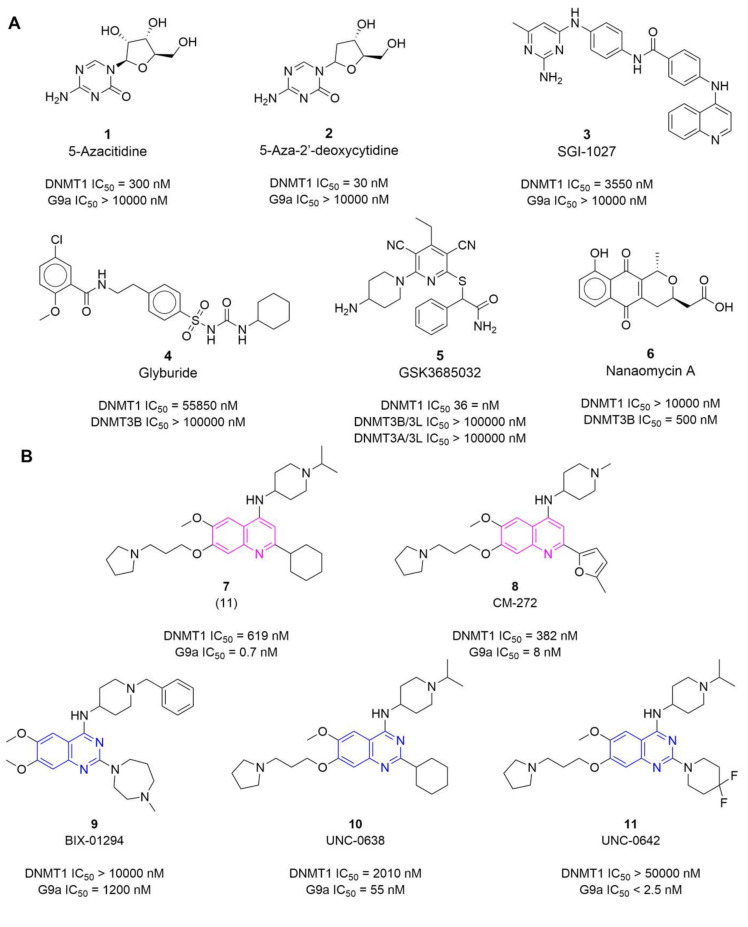
Chemical structures of (**A**) examples of known DNMT inhibitors and compounds with demethylating activity; (**B**) dual DNMT1/G9a inhibitors (quinoline- and quinazoline-based derivatives) reported in the literature. The dual activity profile as available in the literature for epigenetic targets is indicated [27].

Two drugs **1** (5-azacitidine, Vidaza) and **2** (decitabine, 5-aza-2′-deoxycytidine, Dacogen) (Figure 1A) are approved for clinical use to treat the myelodysplastic syndrome [5]. The two drugs are nucleoside analogs that inhibit all three DNMTs (1, 3A, and 3B). Because of the chemical nature of these first-generation DNMT inhibitors (DNMTi), these drugs are characterized by substantial cellular and clinical toxicity, which has driven the development of non-nucleoside, novel, and more specific drugs. Currently, more than 400 non-nucleoside compounds have been tested against at least one DNMT (mostly DNMT1) [20]. Figure 1 shows representative structures of DNMTi and compounds (**1**–**11**) associated with the demethylating activity of DNA. Of note, there is a limited number of selective inhibitors inhibiting DNMTs disclosed so far. For example, **6** (nanaomycin A) (Figure 1A) is a selective inhibitor of DNMT3B (do not inhibit DNMT1) and reactivates silenced tumor suppressor genes in human cancer cells [21]. Recently, **5** (GSK3685032) (Figure 1) was disclosed as the first selective inhibitor of DNMT1 that reduces global methylation, increases expression of target genes, and has antitumor efficacy in acute myeloid leukemia xenograft models [22]. Other related compounds are DNM1-selective inhibitors that reduce global methylation and increases HbF expression, offering the potential for use in treating sickle cell disease [23]. Furthermore, dual inhibitors of DNMT and other epigenetic targets such as G9a and histone deacetylases (HDACs) are emerging as part of a current trend to develop multi-epi-target inhibitors [24,25,26].

Notably, DNMTi have been identified from different sources such as natural products and synthetic small molecule libraries [28,29]. Medicinal chemistry, structure- and ligand-based virtual screening, and high-throughput screening of general screening libraries have led to the identification of DNMTi [30]. There are other drug discovery approaches that are used to identify DNMTi such as de novo design [31] and screening of focused libraries. In this regard, chemical companies are developing screening libraries focused on the most therapeutically relevant epigenetic targets. The chemical samples of the libraries are commercially available for experiential testing. Chemoinformatics contents and chemical diversity analysis of such libraries support their use for drug discovery programs [32].

**Figure 2:**
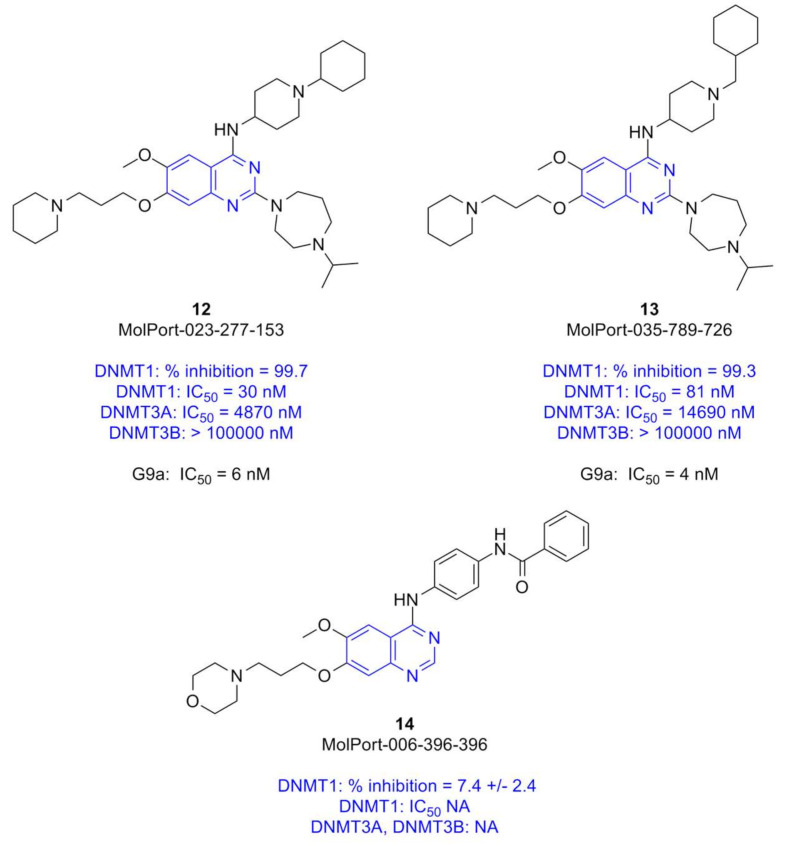
Chemical structures of quinazoline-based derivatives tested in enzymatic inhibition assays. The enzymatic DNMT inhibitory activity measured in this work is included in blue font. Mean value of two measurements. SAH was included as a positive control: IC_50_ (DNMT1) of 0.34 μM; IC_50_ (DNMT3A) of 0.10 μM; (DNMT3B) of 0.03 μM. For reference, the enzymatic G9a inhibitory activity of **12** and **13** [35].

The most promising DNMTi developed so far are molecules with long scaffolds, for example, the 4-aminoquinoline **3** (SGI-1027) and its analogs (Figure 1A) [33]. Other quinoline-based derivatives such as compound **8** (CM-272), where the quinoline ring is the main core scaffold (Figure 1B), have been reported as potent dual inhibitors of DNMT1 and G9a with nanomolar activity [27,34]. Noteworthy, **8** has remarkable in vivo efficacy (70% tumor growth inhibition of a human acute myeloid leukemia xenograft in a mouse model) [27]. The quinolines were developed and further optimized as dual inhibitors based on 7-aminoalkoxy-quinazolines that are inhibitors of G9a such as **12** (MolPort-023-277-153) and **13** (MolPort-035-789-726) (Figure 2) that are potent inhibitors of G9a in enzymatic and in cellular-based assays [35]. Other quinazoline-based derivatives, for example, **9** (BIX-01294), **10** (UNC-0638), and **11** (UNC-0642) (Figure 1B) are potent inhibitors of G9a (at the nanomolar level, in particular **10** and **11** (IC_50_ ≤ 55 nM) [36]. However, the quinazoline-based derivatives reported so far have low DNMT1 potency (>2 μM and are mostly inactive, as shown in Figure 1B). As commented above, DNMTi are emerging as part of programs to develop combination therapies in drug cocktails or compounds targeting multiple epigenetic targets.

Chemical content analysis of the novel epigenetic-focused screening libraries (vide supra) revealed that there are several quinazoline-based derivatives similar to **10** (UNC-0638) and **11** (UNC-0642), including two compounds reported in the literature as inhibitors of G9a. Based on structure-based design reported in the literature and experimental information discussed above [27,35], herein we hypothesized that quinazoline-based derivatives available in the commercial libraries such as **12** and **13** also inhibit the enzymatic activity of DNMTs. 

As part of an ongoing effort to continue expanding the chemical space of DNMTi [31,37], in this work, we report the experimental testing of **12** and **13** and another quinazoline-based derivative (cf. Figure 2) with DNMT1, DNMT3A, and DNMT3B in biochemical enzymatic inhibition assays. We also discuss the molecular dynamics (MD) simulations of the most potent compounds to rationalize their enzymatic inhibitory activity at the molecular level. The findings of this work pave the way to continue exploring quinazoline-based derivatives as inhibitors of DNMTs with multi-epigenetic target activity.

## 2. Methods

### 2.1. Compounds for Experimental Screening

Using a knowledge-based approach, for this work, we selected three quinazoline-based derivatives from epigenetic-focused libraries for experimental testing in enzymatic-based assays (Figure 2). The selection was based on the following criterion:

(1) Knowledge of the promising activity profile of quinoline-based derivatives as dual inhibitors of DNMTs/G9a (vide supra, Figure 1B) in enzymatic and epigenetic functional cellular response;
(2) High structural similarity of the quinoline-based compounds to the selected quinazoline-based derivatives **12** and **13**, that are known to be potent G9a inhibitors in enzymatic and cell-based assays (vide supra, Figure 1B).
(3) Commercial availability of the physical samples from the chemical vendors (Table S3 in the Supplementary Materials).

Based on the rationale explained herein, we hypothesized that the three selected quinazolines in Figure 2 could also inhibit the enzymatic activity of DNMTs.

All three compounds were purchased from MolPort Inc. that confirmed the compound’s purity (in parenthesis): **12** (100%), **13** (98%), and **14** (99.11%). 

### 2.2. Biochemical DNMT Inhibition Assays

The inhibition of the enzymatic activity of DNMT1, DNMT3A, and DNMT3B was tested using the HotSpotSM platform for methyltransferase assays available at Reaction Biology Corporation [38]. HotSpotSM is a low volume radioisotope-based assay that uses tritium-labeled AdoMet (3H-SAM) as a methyl donor. The three test compounds diluted in dimethyl sulfoxide were added using acoustic technology (Echo550, Labcyte, San Jose, CA, USA) into an enzyme/substrate mixture in the nano-liter range. The corresponding reactions were commenced by adding 3H-SAM and incubated at 30 °C. Total final methylations on the substrate (Poly dI-dC in DNMT1, 3A, and 3B assays) were identified by a filter binding method implemented in Reaction Biology. Data analysis was done with Graphed Prism software (La Jolla, CA, USA) for curve fits. The enzymatic inhibition assays were carried out at 1 μM of SAM. In all assays, SAH was used as a standard positive control. The three compounds were tested first with DNMT1 at one 100 μM concentration in duplicate. The most active molecules were tested as DNMT1, DNMT3A, and DNMT3B inhibitors in 10-concentration IC_50_ (effective concentration to inhibit enzymatic activity by 50%) with a threefold serial dilution starting at 100 μM. The research group has recently contracted the screening services of Reaction Biology Corporation to identify a novel DNMTi [37].

### 2.3. Computational Methods

#### 2.3.1. Protein and Ligands Preparation

The crystallographic structure of human DNMT1 (PDB ID: 4WXX) was retrieved from the Protein Data Bank. Available online: https://www.rcsb.org/ (accessed on 9 April 2022) [39]. Missing loops and side-chains were added with YASARA software [40]. The ligands were built and energy-minimized in MOE using the MMFF94x force field. The more stable protomers at physiological pH were identified [41].

#### 2.3.2. Molecular Docking 

Molecular Operating Environment (MOE) software was used to generate the dock conformation of protein-ligand complexes [42]. The grid was centered on the carbon atom of the carboxyl group of GLU 1266 (chain A) with a size of 27 Å^3^ in the presence of the native ligand (SAM). Using the “Triangle Matcher” method, the binding compounds were subjected to 50 search steps (poses) and the default values for the other parameters. The clusters with an RMSD < 2 Å were visually explored. During the docking simulations, the receptor was considered rigid and the ligands flexible. The conformations with the lowest binding energy were selected for an additional MD analysis.

#### 2.3.3. Molecular Dynamics 

MD studies of the protein-ligand complexes were performed using Desmond (version 2021-1, Schrödinger, New York, NY, USA) with the OPLS 2005 forcefield [43]. The most representative docking pose for each ligand was used as a starting point to initiate the MD simulations. The complexes were prepared with the System Builder Utility in a buffered orthomobic box (10 × 10 × 10 Å), using the transferable intermolecular potential with a 3-point model for water (TIP3P). The complexes were neutralized and NaCl was added in a 0.15 M concentration. Complexes were minimized in three stages. In the first stage, water-heavy atoms were restrained with a force constant of 1000 kcal mol^−1^ Å^−2^ (during 100 ps); for the second stage, backbones were constrained with a 10 kcal mol^−1^ Å^−2^ (during 100 ps); and for the third stage, the systems were minimized with no restraints (during 100 ps). The three minimization stages were generated using the default parameters.

The system was submitted to 300 ns of production runs, under NPT ensemble at 1 bar using the Martyna-Tuckerman-Klein (MTK) barostat and 300 K using the Nose–Hoover thermostat. Electrostatic forces were calculated with the smooth PME method using a 9 Å cut-off, while constraints were enforced with the M-SHAKE algorithm. Integration was done every 2 fs, with a recording interval of 50 ps. All protein-ligand complexes were submitted to the “Relax model system” using the default parameters. The quality of the simulation and trajectory analyses were carried out with the tools implemented in the Maestro-GUI (Schrödinger, New York, NY, USA). SAM was used as a procedure control, 150 ns of production were generated using the same protocol described in this section.

## 3. Results and Discussion

### 3.1. Biochemical Inhibition Assays

First, we tested the three compounds **12**, **13**, and **14** (Figure 2) with DNMT1 at a single concentration (100 µM). The results are summarized in Figure 2 and fully detailed in Tables S1 a2 in the Supplementary Materials. Two compounds **12** and **13** showed strong inhibition of DNMT1 (>99%), and **14** showed inhibition of 7.4%. The very low enzymatic inhibitory activity of **14** clearly indicated the need for substitution at position 2 of the quinazoline ring (Figure 2). 

Based on the results at a single concentration, we decided to test the enzymatic inhibitory activity of DNMT1, DNMT3A, and DNMT3B of the two most active compounds **12** and **13** in concentration-response assays. Results, summarized in Figure 2 and detailed in Table S1 in the Supplementary Materials, indicated that **12** is a potent nanomolar and selective inhibitor of DNMT1 (IC_50_ = 30 nM), with higher inhibition of DNMT3A (IC_50_ = 4870 nM) and no enzymatic inhibition of DNMT3B (IC_50_ > 100,000 nM). Notably, under the assay conditions used in this work, **12** was about ten times more potent against DNMT1 than the positive control SAH (IC_50_ = 340 nM). The structural analog **13** had a similar inhibitory activity profile with similar inhibitory potency but higher selectivity towards DNMT1 (IC_50_ = 81 nM) over DNMT3A (IC_50_ = 14,690 nM), and DNMT3B (IC_50_ > 100,000 nM) (Figure 2 and Table S1).

The nanomolar enzymatic inhibitory potency of DNMT1 of both 7-aminoalkoxy-quinazolines **12** and **13** is about ten times that of the positive control SAH. Despite the high variability across DNMT inhibitory assays and the challenge to reproduce the IC_50_ values accurately across different laboratories, there are few compounds reported in the literature with low nanomolar inhibition of DNMT1 [44]. For instance, in one of the most recent studies testing different DNMTi under the same assay conditions, **5** (GSK3685032) strongly inhibited DNMT1 (IC_50_ = 30 nM) and did not inhibit DNMT3A and 3B (Figure 1A). In that work, Pappalardi et al., also tested the well-known pan inhibitor **3** (SGI-1027), showing IC_50_ values of 1030 nM (DNMT1); 13,000 nM (DNMT3A), and 7000 nM (DNMT3B) [22]. In the work of Pappalardi et al., SAH (also used as a reference) had IC_50_ values of 540 nM, 100 nM, and 90 nM for DNMT1, DNMT3A, and DNMT3B, respectively. Such values for the positive control generally agree with the IC_50_ values measured under the assay conditions used in this work (340 nM, 100 nM, and 30 nM for DNMT1, DNMT3A, and DNMT3B, respectively, Table S2 in the Supplementary Materials).

In addition to the promising DNMT enzymatic inhibition profile of compounds **12** and **13**, it is remarkable the reported high inhibition of both molecules of the epigenetic target G9a (IC_50_ = 6, and 4 nM, respectively, Figure 2) [35]. As discussed in the Introduction, based on the structural relationship between the quinazolines tested in this work with the 4-aminoquinolines reported as dual inhibitors of G9a and DNMT1 [27], we hypothesized that quinazolines such as **12** and **13**, could inhibit DNMT1. Biochemical inhibition assays reported in this work confirmed the hypothesis. Further testing with DNMT3A and DNMT3B revealed that both molecules are potent inhibitors of DNMT1 and selective versus DNMT3A and DNMT3B. Thus far, other than **5** (GSK3685032), the two quinazolines reported in this work are the few small-molecule selective inhibitors of DNMT1 over DNMT3A and DNMT3B.

### 3.2. Molecular Docking and Dynamics Simulations with DNMTs

Computational methods have been used to identify novel inhibitors, optimize their activity, and/or to further understand their activity at the molecular level of compound targeting epigenetic targets, including DNMTs. These methods are collectively referred to as “epi-informatics” [30]. As previously discussed, computational methods are not used necessary to predict or identify novel inhibitors but the computational studies provide key insights to study the mechanism of action and rationalize the activity of active molecules at the molecular level. For example, we recently conducted a molecular and activity landscape modeling study to rationalize the reported enzymatic inhibitory activity of 251 G9a inhibitors [34], and 50 4-aminoquinolines as dual inhibitors of G9a and DNMT1 [45]. Results of that work yielded the establishment of a robust structural hypothesis of protein-ligand interactions associated with the dual activity or selectivity with the epigenetic targets.

In this work, we employed molecular docking and dynamics simulations to provide insights into the DNMTs enzymatic inhibitory activity of **12** and **13** at the structural level. For this purpose, we took advantage of the availability of the three-dimensional structural information of DNMT1, DNMT3A, and DNMT3B.

**Figure 3:**
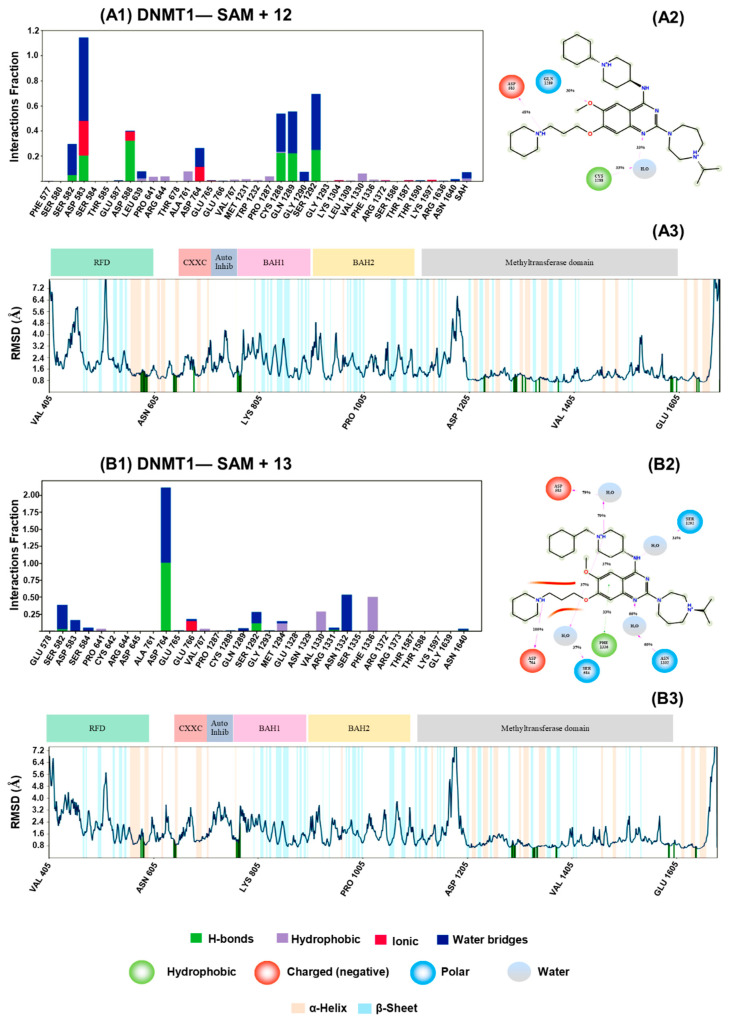
Molecular dynamic results of compounds **12** and **13** against DNMT1. 300 ns were produced peer each compound. The (**A1**,**B1**) panels show the interactions between ligands and the DNMT1 structure in the presence of *S*-adenosyl-*L*-methionine (SAM). Additionally, panels (**A2**,**B2**) show the representative key interaction during the last 30 ns of molecular dynamic productions. Finally, panels (**A3**,**B3**) illustrate the conformational changes (RMSD values) on different key domains on DNMT1.

Figure 3 summarizes the interactions between **12** and **13** and DNMT1, according to the MD simulations. The conserved interactions with SER 582, ASP 764, and SER 1292 in both compounds. However, the generation of stable interactions with ASP 583, ASP 588, CYS 1288, and GLN 1289 (Figure 3(A1,A2)). Interestingly, **13** has been distinguished by other key interactions (GLU 766, VAL 1330, ASN 1332, and PHE 1336). Despite the differences in key interactions, the compounds studied tend to establish the conformation (reducing the RMSD values) of CXXC (647–691 aa) and autoinhibition (699–733 aa) domains of DNMT1 (Figure 3(A3,B3)), in relationship with SAM (Figure S1C) in the Supplementary Materials). We emphasize that these domains are present only in DNMT1 and are not its 3A or 3B isoforms [46], which could explain the selective enzymatic inactivation of DNMT1 [47]. Additionally, we analyze the specific conformational changes associated with the interaction of **12** and **13** against DNMT1 (Figure S2 in the Supplementary Materials).

Recently, we described at the structural level the conformational changes generated on G9a in their SET domain by the interaction with quinoline-based compounds [34]. This work describes the conformational changes associated with the interaction of quinoline-based compounds against the CXXC and autoinhibition domain on DNMT1.

Several groups are working identifying novel potent DNMTi, including dual inhibitors of epigenetic writers such as DNMT and G9a (vide supra). For example, we recently reported DNMT1 inhibitors with novel chemical scaffolds, including two approved for clinical use. However, those compounds lack enough potency. For instance, **4** showed and IC_50_ = 55.85 μM using the same assay conditions used in this work [37]. The knowledge of the promising enzymatic and cell-based activity profile of quinoline-based compounds such as **8** (Figure 1B) as dual inhibitors of DNMT1 and G9a; the high structural similarity of the quinoline-based derivatives to the quinazoline-based derivatives with strong inhibition profile of G9a; plus the commercial availability of quinazoline-based derivatives in epigenetic focused libraries led us to identifytwo 7-aminoalkoxy-quinazolines with 30 and 81 nM potency toward DNMT1 (Figure 2). Molecular modeling studies suggest that the selective inhibition of DNMT1 was carried out by the induced conformational change on their CXXC and autoinhibition domains, which is essential for enzymatic activity [47].

Notably, it is reported in the literature that **12** and **13** have high in vitro potency versus G9a (IC_50_ = 6 and 4 nM, respectively) and are also highly potent in reducing H3K9me2 levels in human breast adenocarcinoma (MDA-MB-231) cells (26 and 25 nM, respectively) and with low cell toxicity (EC_50_ of 3.3 and 2.8 μM, respectively) [35]. Potting together, the two 7-aminoalkoxy-quinazolines **12** and **13** are promising compounds to continue developing as polypharmacological, specifically, dual-epigenetic target inhibitors as candidates compounds with potential therapeutic applications.

In silico target profiling of the three compounds tested in this work (Figure 2), with the recently developed online web server Epigenetic Target Profiler [48], suggest that all compounds could be active with additional epigenetic targets such as HDACs (Figure S3 in the Supplementary Materials). It would remain to continue exploring computationally (e.g., using structure-based methods) and then experimentally the predicted activity of the molecules.

## 4. Conclusions

We identified two 7-aminoalkoxy-quinazolines: **12** and **13**, with nanomolar inhibition of DNMT1 (30 nM y 80 nM, respectively) in enzymatic inhibition assays. Notably, both compounds showed better inhibitory activity than the positive control, SAH (340 μM), less inhibition of DNM3A (4.87 and 14.69 μM) and none of them inhibited DNMT3B. Also, these have been reported as potent inhibitors of another epigenetic target writer, G9a, in enzymatic and human breast adenocarcinoma (MDA-MB-231) cell-based assays with low cell toxicity. It remains to demonstrate that **12** and **13** are also able to reduce methylation levels in cell-based assays. However, their high structural similarity to the quinoline-based derivatives such as **CM-272** (Figure 1B), which are known to effectively reduce DNA methylation in cell-based assays, strongly support the hypothesis that the quinazolines reported in this work will also be inhibitors in cell based-assays. Such a hypothesis will be tested and reported in due course. The two active compounds identified in this work are structurally similar with conserving key interactions against SER 582, ASP 764, and SER1292 on DNMT1 in presence of their cofactor SAM. The molecular dynamics results suggest that the DNMT1 inhibition of **12** and **13** is caused by the conformational changes on the CXXC and autoinhibition domains. From the mechanistic point of view, one of the main perspectives of this study is to test if the quinazoline-based derivatives; for instance, **12** is DNA—substrate competitive. This will be done through enzymatic DNA-substrate competitive assays. The results will be reported in due course. In all, the results of this work are a contribution towards the further investigation and development of DNMTi as part of multiple epigenetic target therapies.

## Data Availability

Not applicable.

## Supplementary Materials

The following Supplementary Materials can be downloaded at: https://www.mdpi.com/article/10.3390/molecules27092892/s1, Table S1: Number of quinazolines and 7-aminoalkoxy-quinazolines present in various epigenetic focused libraries; Table S2: Results of the relative enzymatic activity of DNMT1 as percentages^a^; Table S3: Results of concentration-response assays for selected quinazolines (IC50) with DNMT1, DNMT3A and DNMT3B^a^; Figure S1: Analysis of 150 ns production of the molecular dynamics of production S-Adenil-Metione (SAM) against DNMT1; Figure S2: Final conformational changes on DNMT1 from molecular dynamics calculations; Figure S3: In silico profiling of the three compounds with the free online and validate server Epigenetic Target Profiler.

## Sample Availability

Samples of the compounds are not available from the authors.
