# Supplementary material for: 7-Aminoalkoxy-Quinazolines from Epigenetic Focused Libraries Are Potent and Selective Inhibitors of DNA Methyltransferase 1
Source: Molecules. 2022 Apr 30;27(9):2892. doi: 10.3390/molecules27092892 (PMC9102847; doi:10.3390/molecules27092892)
Supplement: Supplementary file 1 [file molecules-27-02892-s001.zip › molecules-1698251-SI.pdf]

SUPPORTING INFORMATION

7-Aminoalkoxy-Quinazolines from Epigenetic Focused Libraries are Potent and Selective Inhibitors of DNA Methyltransferase 1

José L. Medina-Franco[1,*], Edgar López-López[1,2], Liliam P. Martínez-Fernández[1]

[1] *DIFACQUIM research group, Department of Pharmacy, School of Chemistry, National Autonomous University of Mexico, Avenida Universidad 3000, Mexico City 04510, Mexico.*

[2] *Department of Pharmacology, Center for Research and Advanced Studies of the National Polytechnic Institute (CINVESTAV), Avenida Instituto Politécnico Nacional 2508, Mexico City 07360, Mexico.*

**Corresponding author. Email: medinajl@unam.mx*

Contents

		Page
**Table S1**	Number of quinazolines and 7-aminoalkoxy-quinazolines present in various epigenetic focused libraries.	S2
**Table S2**	Results of the relative enzymatic activity of DNMT1 as percentages [a].	S3
**Table S3**	Results of dose-response evaluations for selected quinazolines (IC_{50}) with DNMT1, DNMT3A and DNMT3B [a].	S3
**Figure S1**	Analysis of 150 ns production of the molecular dynamics of production S-Adenil-Metione (SAM) against DNMT1.	S4
**Figure S2**	Final conformational changes on DNMT1 from molecular dynamics calculations.	S5
**Figure S3**	*In silico* profiling of the three compounds with the free online and validate server Epigenetic Target Profiler.	S6

Table S1. Number of quinazolines and 7-aminoalkoxy-quinazolines present in various epigenetic focused libraries.

DATABASE	QUINAZOLINES	7-AMINOALKOXY-QUINAZOLINES
ASINEX	4	
AXON	8	7
CHEMDIV	559	
ENAMINE	61	
TARGETMOL	16	12
SELLECK	19	10
OTAVA_DNMT1	5	
OTAVA_DNMT3B	17	1
ENZO	1	1
LIFECHEM	18	
TOTAL	708	31

Table S2. Results of the relative enzymatic activity of DNMT1 as percentages [a].

COMPOUND	DNMT1
**MOLPORT-023-277-153**	0.3 ± 0.10
**MOLPORT-035-789-726**	0.7 ± 0.02
**MOLPORT-006-396-396**	92.6 ± 2.40

[a] Mean value of two measurements ± standard deviation.

Table S3. Results of concentration-response assays for selected quinazolines (IC_{50}) with DNMT1, DNMT3A and DNMT3B [a].

COMPOUND	DNMT1 (IC_{50} MM)	DNMT3A (IC_{50} MM)	DNMT3B (IC_{50} MM)
**MOLPORT-023-277-153**	0.030 (± 0.016)	4.87 (± 0.47)	> 100 μM
**MOLPORT-035-789-726**	0.081 (± 0.029)	14.69 (± 0.56)	> 100 μM

[a] Mean value of two measurements ± standard deviation. SAH was included as a positive control: IC_{50} (DNMT1) of 0.34 μM; IC_{50} (DNMT3A) of 0.10 μM; (DNMT3B) of 0.03 μM.

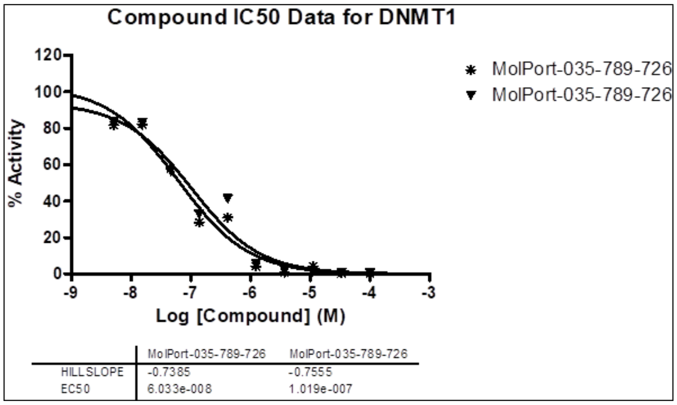

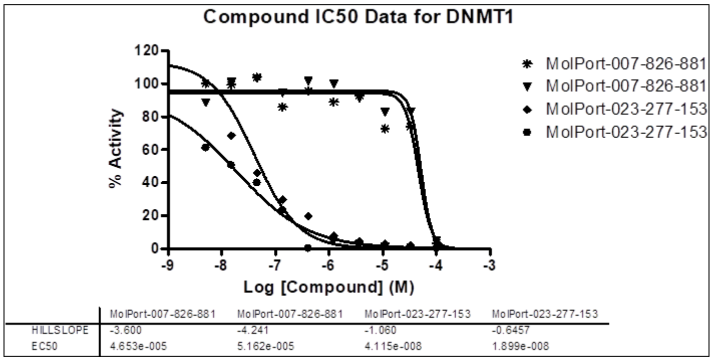

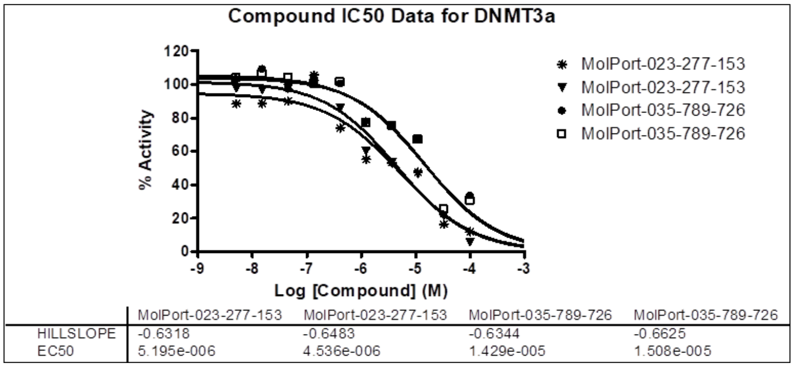

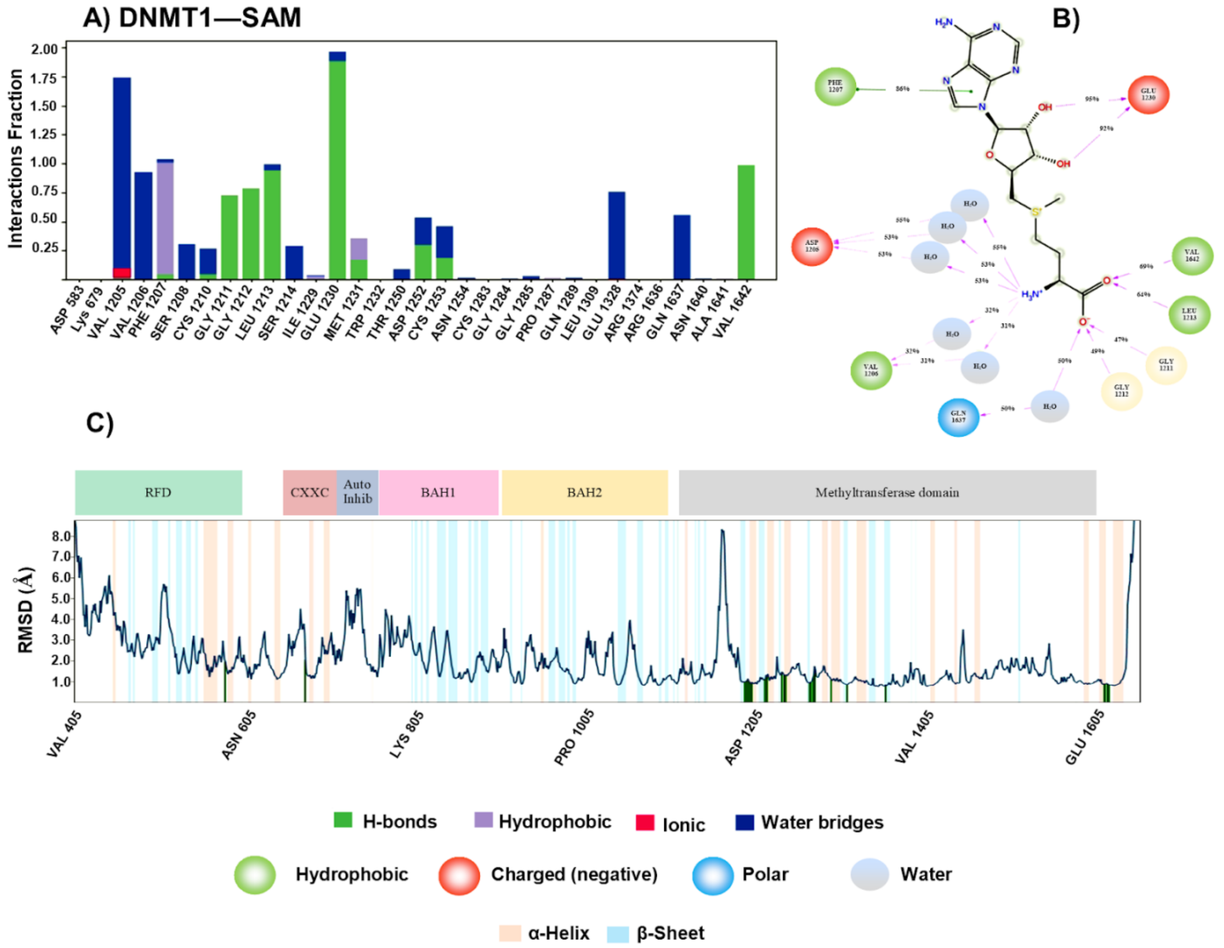

Figure S1. Analysis of 150 ns production of the molecular dynamics of production S-Adenil-Metione (SAM) against DNMT1. **A)** Molecular dynamic interactions of SAM against DNMT1. **B)** Molecular dynamic interactions of SAM against DNMT1 during the last 30ns of production. **C)** Conformational changes observed during the molecular dynamics of SAM against DNMT1.

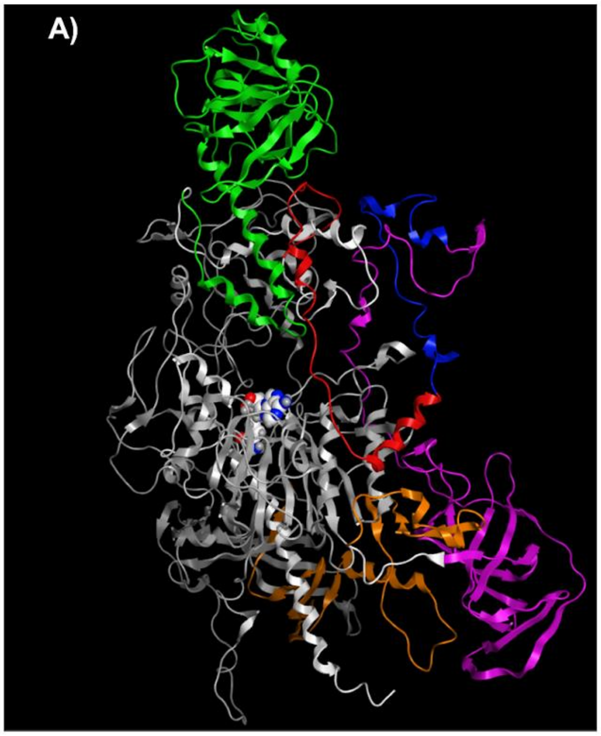

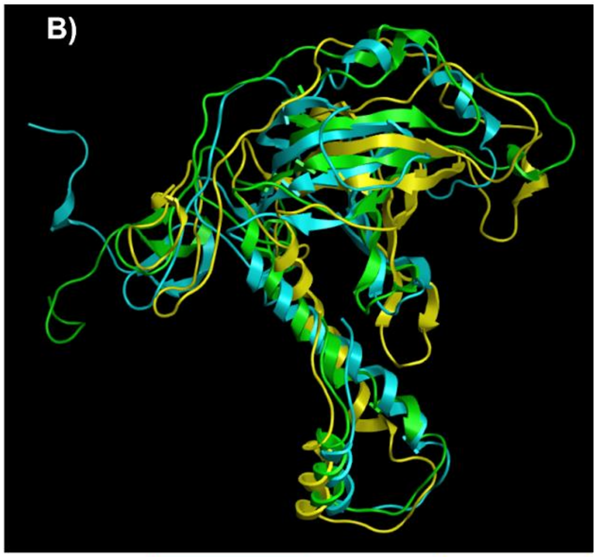

RFD	SAM	SAM + 12	SAM + 13
SAM	0.0		
SAM + 12	6.31	0.0	
SAM + 13	8.38	7.59	0.0

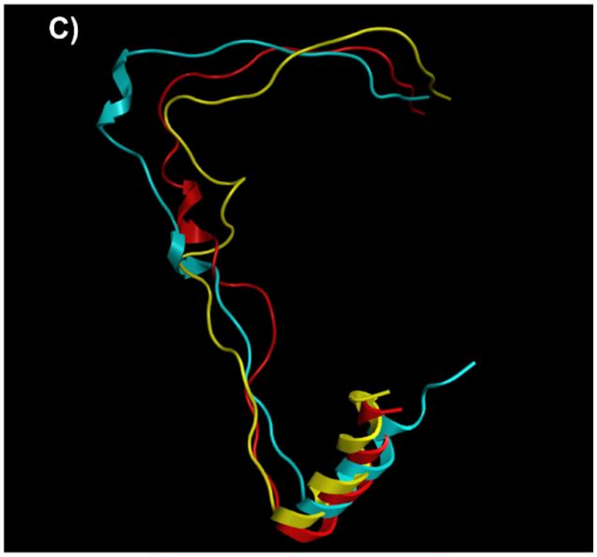

CXXC	SAM	SAM + 12	SAM + 13
SAM	0.0		
SAM + 12	4.82	0.0	
SAM + 13	4.17	6.14	0.0

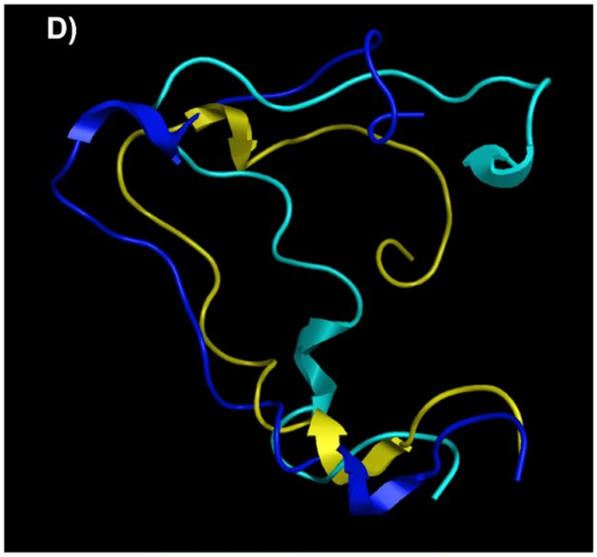

Auto Inhib	SAM	SAM + 12	SAM + 13
SAM	0.0		
SAM + 12	4.82	0.0	
SAM + 13	4.17	6.14	0.0

Figure S2. Final conformational changes on DNMT1 from molecular dynamics calculations. **A)** DNMT1-SAM model. The RFD (green), CXXC (red), autoinhibition (navy blue), BAH1 (pink), BAH2 (orange), and DNA-methyl transferase (gray) domains were shown. Panels **B), C),** and **D)** illustrate the conformational changes (with RMSD values) on the RFD, CXXC, and autoinhibition domains of DNMT1 in presence of SAM, SAM + **12,** and SAM + **13**, respectively.

Epigenetic Target Profiler

MolPort-023-277-153

Name	ChEMBL ID	Gene	Status	Quartile
Histone-lysine N-methyltransferase, H3 lysine-9 specific 3	CHEMBL6032	EHMT2	Known	
Serine-protein kinase ATM	CHEMBL3797	ATM	Predicted	Q4
Serine/threonine-protein kinase Aurora-A	CHEMBL4722	AURKA	Predicted	Q4
Serine/threonine-protein kinase Aurora-B	CHEMBL2185	AURKB	Predicted	Q4
Bromodomain-containing protein 2	CHEMBL1293289	BRD2	Predicted	Q4
Histone-arginine methyltransferase CARM1	CHEMBL5406	CARM1	Predicted	Q4
Cyclin-dependent kinase 7	CHEMBL3055	CDK7	Predicted	Q4
CREB-binding protein	CHEMBL5747	CREBBP	Predicted	Q4
Histone deacetylase 1	CHEMBL325	HDAC1	Predicted	Q4
Histone deacetylase 6	CHEMBL1865	HDAC6	Predicted	Q4
Histone deacetylase 8	CHEMBL3192	HDAC8	Predicted	Q4
Lysine-specific demethylase 4C	CHEMBL6175	KDM4C	Predicted	Q4
Lysine-specific demethylase 5A	CHEMBL2424504	KDM5A	Predicted	Q4
Poly [ADP-ribose] polymerase-1	CHEMBL3105	PARP1	Predicted	Q4

MolPort-035-789-726

Name	ChEMBL ID	Gene	Status	Quartile
Histone-lysine N-methyltransferase, H3 lysine-9 specific 3	CHEMBL6032	EHMT2	Known	
Serine-protein kinase ATM	CHEMBL3797	ATM	Predicted	Q4
Serine/threonine-protein kinase Aurora-A	CHEMBL4722	AURKA	Predicted	Q4
Serine/threonine-protein kinase Aurora-B	CHEMBL2185	AURKB	Predicted	Q4
Bromodomain-containing protein 2	CHEMBL1293289	BRD2	Predicted	Q4
Histone-arginine methyltransferase CARM1	CHEMBL5406	CARM1	Predicted	Q4
Cyclin-dependent kinase 7	CHEMBL3055	CDK7	Predicted	Q4
CREB-binding protein	CHEMBL5747	CREBBP	Predicted	Q4
Histone deacetylase 1	CHEMBL325	HDAC1	Predicted	Q4
Histone deacetylase 8	CHEMBL3192	HDAC8	Predicted	Q4
Lysine-specific demethylase 4C	CHEMBL6175	KDM4C	Predicted	Q4
Lysine-specific demethylase 5A	CHEMBL2424504	KDM5A	Predicted	Q4
Poly [ADP-ribose] polymerase-1	CHEMBL3105	PARP1	Predicted	Q4
Protein kinase N1	CHEMBL3384	PKN1	Predicted	Q4
DNA-dependent protein kinase	CHEMBL3142	PRKDC	Predicted	Q4

MolPort-006-396-396

Name	ChEMBL ID	Gene	Status	Quartile
Serine/threonine-protein kinase Aurora-A	CHEMBL4722	AURKA	Known	
Serine/threonine-protein kinase Aurora-B	CHEMBL2185	AURKB	Known	
Serine-protein kinase ATM	CHEMBL3797	ATM	Predicted	Q1
Histone-arginine methyltransferase CARM1	CHEMBL5406	CARM1	Predicted	Q2
Histone deacetylase 1	CHEMBL325	HDAC1	Predicted	Q2
Histone deacetylase 3	CHEMBL1829	HDAC3	Predicted	Q2
CREB-binding protein	CHEMBL5747	CREBBP	Predicted	Q3
Histone deacetylase 6	CHEMBL1865	HDAC6	Predicted	Q3
Histone deacetylase 8	CHEMBL3192	HDAC8	Predicted	Q3
Lysine-specific demethylase 4C	CHEMBL6175	KDM4C	Predicted	Q3
Lysine-specific demethylase 5A	CHEMBL2424504	KDM5A	Predicted	Q3
Bromodomain-containing protein 2	CHEMBL1293289	BRD2	Predicted	Q4

Figure S3. *In silico* profiling of the three compounds with the free online and validate server Epigenetic Target Profiler. EHMT2 = G9a. The Quartile column indicates the confidence in the prediction (higher values indicate higher confidence. See details in the reference: Sánchez-Cruz. N. et al. J. Med. Chem. 2021, 64:8208-8220.
